# Generation of Helper Plasmids Encoding Mutant Adeno-associated Virus Type 2 Capsid Proteins with Increased Resistance against Proteasomal Degradation

**Published:** 2013-07

**Authors:** Naghmeh Ahmadiankia, Vajiheh Neshati, Zeinab Neshati, Jim Swildens, Antoine AF de Vries

**Affiliations:** 1 Shahroud University of Medical Sciences, Shahroud, Iran; 2 Stem Cell and Regenerative Medicine Research Department, Iranian Academic Center for Education, Culture and Research (ACECR), Mashhad Branch, Mashhad, Iran; 3 Department of Cardiology, Leiden University Medical Center, Leiden, the Netherlands

**Keywords:** Gene therapy, Viral vector, Adeno-associated virus type 2, Capsid protein, Proteasomal degradation, Mutagenesis, Transduction efficiency

## Abstract

***Objective(s):*** Adeno-associated virus type 2 (AAV2) vectors are widely used for both experimental and clinical gene therapy. A recent research has shown that the performance of these vectors can be greatly improved by substitution of specific surface-exposed tyrosine residues with phenylalanines. In this study, a fast and simple method is presented to generate AAV2 vector helper plasmids encoding capsid proteins with single, double or triple Y→F mutations.

***Materials and Methods:*** A one-step, high-fidelity polymerase chain reaction (PCR) cloning procedure involving the use of two partially overlapping primers to amplify a circular DNA template was applied to produce AAV2 *cap* genes encoding VP1 mutants with Y→F substitutions in residues 444, 500 or 730. The resulting constructs were used to make the different double and triple mutant by another round of PCR (Y444500F mutant), subcloning (Y444730F and Y500730F mutants) or a combination of both techniques (Y444500730F mutant).

***Results:*** Nucleotide sequence analysis revealed successful introduction of the desired mutations in the AAV2 *cap* gene and showed the absence of any unintended mutations in the DNA fragments used to assemble the final set of AAV2 vector helper plasmids. The correctness of these plasmids was further confirmed by restriction mapping.

***Conclusion:*** PCR-based, single-step site-directed mutagenesis of circular DNA templates is a highly efficient and cost-effective method to generate AAV2 vector helper plasmids encoding mutant Cap proteins for the production of vector particles with increased gene transfer efficiency.

## Introduction

Recombinant adeno-associated virus type 2 (AAV2) vectors have been used in clinical trials for the treatment of different diseases (1-3). However, in many cases, relatively large vector doses are required to obtain a therapeutic effect. The requirement of large vector doses imposes several problems like the technical difficulty to produce sufficient vector particles and the immune responses elicited by high numbers of these particles (4). One way to reduce these problems is through the development of AAV vectors with increased transduction efficiencies. The transduction efficiency of viral vectors is determined by many factors including (i) the availability of receptors on the surface of the target cells, (ii) the route of intracellular trafficking and (iii) the efficiency with which vector genomes are converted into transcriptionally active templates (5, 6). Previous research showed that AAV2 vector-mediated gene transfer in mammalian cells is limited by the ubiquitination of internalized AAV capsids and their subsequent degradation in proteasomes (7). This limitation can be partially overcome in some cell types but not in others by performing the transduction process in the presence of proteasome or ubiquitin ligase inhibitors (8). Prolonged exposure to these inhibitors is, however, poorly tolerated by many cell types. It has also been documented that epidermal growth factor receptor protein tyrosine kinase (EGFR-PTK) signaling induces ubiquitination of AAV2 capsids and inhibits the nuclear transport of AAV vectors by promoting their proteasome- mediated degradation (9).

It was subsequently shown that EGFR-PTK can phosphorylate AAV2 capsids at tyrosine residues. Tyrosine-phosphorylated AAV2 vectors enter cells as efficiently as their non-phosphorylated counterparts but have a reduced capacity to transduce cells, most likely as a result of the ubiquitination and subsequent proteasome-mediated degradation of their capsids (10). Consistently, recent studies have demonstrated that substitution of certain surface-exposed tyrosine residues with phenylalanines allows AAV2 vectors to escape phosphorylation and subsequent ubiquitination. This causes a reduction in the proteasome-mediated degradation of internalized recombinant AAV2 particles leading to an increase in the transduction efficiency of AAV2 vectors (11-13).

Inspection of the surface structure of AAV2 capsids, revealed a total of 7 surface-exposed tyrosine residues (*i.e.* Y252, Y272, Y444, Y500, Y700, Y704 and Y730) (10). Previous experiments showed that changing the tyrosine residues at positions 444, 500 or 730 of the largest capsid protein (*i.e.* VP1) to the non-phosphorylatable phenylalanine residues could increase the specific gene transfer activity of AAV2 vectors in many cell types. Combining these mutations in single capsids often enhanced the transduction efficiency of AAV2 vectors (11-13) further. These findings prompted us to develop a simple and robust method to generate AAV2 *cap *gene mutants quickly.

## Materials and Methods

***Construction of plasmid pUCBM21.Cap2***

Plasmid pUCBM21.Cap2 (5282 bp) was used as template for substitution mutagenesis. This construct was generated by combining the 2.6-kb AAV2 *cap* gene-containing HindIII × (filled-in) ClaI fragment of AAV2 vector helper plasmid pDG (14) with the 2.7-kb HindIII × SmaI fragment of plasmid pUCBM21PSXSP. pUCBM21PSXSP was derived from plasmid pUCBM21 (Boehringer Mannheim) in two cloning steps. First, the hybridization product of oligodeoxyribonucleotides 5’ GATCCCGGGATTTAAATGTTTAAACG 3’ and 5’ AATTCGTTTAAACATTTAAATCCCGG 3’ was inserted in between the BamHI and EcoRI recognition sites of pUCBM21 to make pUCMB21SP. Next, the annealing product of oligodeoxyribonucleotides 5’ AGCTTGTTTAAACATTTAAATCTCGAGCCATGGAT 3’ and 5’ ATCCATGGCTCGAGATTTAAATGTTTAAACA 3’ was inserted between the HindIII and EcoRV recognition sequences of pUCMB21SP to generate pUCBM21PSXSP. DNA constructions were carried out with enzymes from either Fermentas or New England Biolabs using established procedures (15) or following the instructions provided with specific reagents. Plasmids were propagated in *Escherichia -coli* strains GeneHogs or DH5α (both from Life Technologies). Large-scale plasmid DNA purifications were performed using the JETSTAR 2.0 Maxi Kit (GENOMED).

***Construction of plasmids pUCBM21.Cap2Y444F, pUCBM21.Cap2Y500F and pUCBM21.Cap2Y730F by polymerase chain reaction (PCR)-based site-directed mutagenesis***

*Preparation of PCR mixtures*

PCR mixtures were prepared in thin-walled 0.2 ml microtubes (Eppendorf) and consisted of 5 μl of template DNA, (± 1 ng/µl), 2 μl of 10 µM forward primer and 2 μl of 10 µM reverse primer, 5 μl of 2.5 mM each dNTP, 10 μl 5 X Hi-Fi Reaction Buffer (Bioline), 1.5 μl of 3% dimethyl sulphoxide (Bioline) and either 1 μl of 1U/µl VELOCITY DNA polymerase and 23.5 μl water (tubes 1, 3 and 5) or 24.5 µl water (tubes 2, 4 and 6). Tubes 1 and 2 contained primers A001 and A002 while primers A003 and A004 were added to tubes 3 and 4 and tubes 5 and 6 contained primers A005 and A006 (Table 1).

*PCR cycling conditions*

PCR mixtures were transferred from ice to a Biometra TGradient 96 PCR machine preheated to 98°C (hot start) and incubated for 2 min at this temperature to pre-denature the template DNA. This was followed by 20 cycles of 30 sec at 98°C (template denaturation), 30 sec at 48°C (primer annealing) and 4 min at 72°C (primer extension). PCR procedure was completed by a final cycle of 30 sec at 98°C, 30 sec at 40°C and 8 min at 72°C.

*Purification of PCR products*

PCR amplification products were purified using SureClean (Bioline) to remove dNTPs, primers and DNA polymerase molecules.

**Table 1:** Nucleotide sequences of primers used for PCR mutagenesis. Underlined nucleotides represent overlaps between forward and reverse primers. Point mutations leading to Y→F substitution are indicated in boldface. The primer pairs were designed in such a manner that the non-overlapping parts of the forward and reverse primer bound more strongly to the template DNA comparing to when both primers bound with their overlapping parts to each other. This particular primer design strongly reduces primer dimer formation during PCR in comparison with the use of fully complementary forward and reverse primers. Moreover, it allows amplification of nicked DNA templates generated during earlier PCR cycles (Figure 1) (16).

Primer	Sequence (5'→3')
A001 (Cap2Y444F-F)	CTGTATT **T** CTTGAGCAGAACAAACACTCCAAGTGGAAC (38 bp)
A002 (Cap2Y444F-R)	CTGCTCAAG **A** AATACAGGTACTGGTCGATGAGAGGATTC (39 bp)
A003 (Cap2Y500F-F)	GTGAAT **T** CTCGTGGACTGGAGCTACCAAGTACCACC (36 bp)
A004 (Cap2Y500F-R)	GTCCACGAG **A** ATTCACTGTTGTTGTTATCCGCAGATG (37 bp)
A005 (Cap2Y730F-F)	ACCAGAT **T** CCTGACTCGTAATCTGTAATTGCTTGTTAATCAA (42 bp)
A006 (Cap2Y730F-R)	AGTCAGG **A** ATCTGGTGCCAATGGGGCGAG (29 bp)

**Figure 1:**
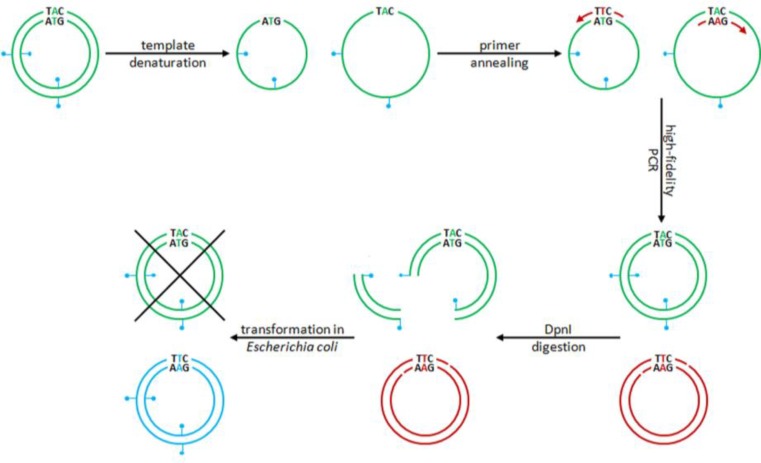
Schematic diagram of the PCR-based, site-directed mutagenesis procedure used to produce AAV2 vector packaging plasmids encoding capsid proteins with increased resistance against proteasomal degradation. Following synthesis of mutant DNA strands, PCR mixtures are subjected to DpnI digestion to selectively degrade the bacterial input DNA, which differs from the newly synthesized PCR products by being methylated at A residues in the DpnI recognition sequence (*i.e.* 5’ GATC 3’). Red circle, “old” template DNA; blue pedal, methyl group; green arrow, PCR primer; green circle, “new” PCR product; blue circle, mutant DNA recovered from bacteria

*Fragmentation of DNA template*

Purified amplification products were dissolved in 40 µl of 1X Tango/Y^+^ buffer (Fermentas). Subsequently, 1 µl of 10 U/µl of DpnI was added and samples were incubated for 4 hr at 37°C. DpnI only cleaves the DNA which is modified by the bacterial enzyme deoxyadenosine methyltransferase (Dam). The DpnI treatment thus causes selective degradation of the methylated parental DNA leaving the newly synthesized mutant DNA intact (Figure 1). The DpnI was inactivated by incubation for 20 min at 80°C.

*Bacterial transformation*

After DpnI digestion, 45 μl of chemocompetent Gene Hogs cells were added to 5 μl of each DpnI-treated PCR sample. GeneHogs cells were transformed by the process of heat-shocking. After recovery for 1 hr at 37°C in 300 μl Luria-Bertani medium (LB) without antibiotics, transformed cells were spread on LB agar plates containing 100 μg/ml ampicillin (Roche Applied Science). Agar plates were incubated overnight at 37°C. The next day, two colonies from each plate were used to inoculate two separate 2-l Erlenmeyer flaks containing 250 ml of LB with 50 μg/ml ampicillin. Bacterial cultures were incubated overnight at 37°C in an incubator-shaker (Innova 44, Eppendorf).

*Large-scale DNA purification*

The bacteria in each culture were pelleted by centrifugation for 10 min at 6000 rpm in a Sorvall RC-26 PLUS Superspeed centrifuge equipped with an SLA-3000 rotor. Each bacterial pellet was subjected to large-scale DNA purification using the aforementioned JETSTAR 2.0 Maxi Kit. The resulting constructs were designated pUCBM21.Cap2Y444F, pUCBM21.Cap2Y500F and pUCBM21.Cap2Y730F for the amplification products of primers A001 and A002, A003 and A004 and A005 and A006, respectively.

*Restriction mapping*

Before subjecting the plasmid DNAs to nucleotide sequence analysis, their integrity and global structure were investigated by restriction endonuclease digestion using the enzymes ApaLI, ApaI, AvaI, BglI and DraI. The digestion products were analyzed by conventional agarose gel electrophoresis using the Tris-acetate-EDTA buffer system (15). The gel images were archived with a ChemiDoc XRS Imaging System and Quantity One Software (both from Bio-Rad).

*Nucleotide sequence analysis*

To check whether the AAV2 *cap *sequences in each plasmid contained the desired A-T transversion and did not possess any additional, unintended mutations, nucleotide sequence analysis was performed. To this end, 750 ng of plasmid DNA was mixed with 1 μl of 10 μM sequencing primer (Table 2) and supplemented with water to a total volume of 15 μl. DNA sequencing was carried out by Leiden Genome Technology Center (http://www.lgtc.nl/) using a BigDye Terminator v3.1 Cycle Sequencing Kit and a 3730xl DNA Analyzer (both from Applied Biosystems).

*Nucleotide sequencing analysis*

Sequencing results were analysed using SnapGene Viewer(http://www.snapgene.com/products/snapgene_viewer/) and EMBOSS Needle for the pairwise alignment of nucleotide sequences (http://www.ebi.ac.uk/Tools/services/web/toolform.ebi?tool=emboss_needle&context=nucleotide).

*Construction of plasmid pUCBM21.Cap2Y444500F*

Due to the absence of convenient restriction enzyme recognition sites in the part of the AAV2 *cap* gene flanked by the codons for amino acid residues 444 and 500, construct pUCBM21.Cap2Y444500F encoding the Cap double mutant Y444500F was generated from plasmid pUCBM21.Cap2Y444F with primers A003 and A004 and from plasmid pUCBM21.Cap2Y500F using primers A001 and A002 as described above.

*Construction of plasmids pUCBM21.Cap2Y444730F, pUCBM21.Cap2Y500730F and pUCBM21.Cap2 Y444500730F*

ConstructspUCBM21.Cap2Y444730F,pUCBM21.Cap2Y500730F and pUCBM21.Cap2 Y444500730F were made by combining the 1.1-kb SspI × XcmI fragment of pUCBM21.Cap2Y730F with the 4.2 kb SspI × XcmI fragments of pUCBM21.Cap2Y444F, pUCBM21.Cap2Y5000F and pUCBM21.Cap2Y444500F, respectively. The DNA fragments were isolated from agarose gel with the aid of the JETSORB Gel Extraction Kit (GENOMED) based on the instructions of the manufacturer. Ligations were done using bacteriophage T4 DNA ligase. The correctness of the cloning products was verified by restriction analysis with five different enzymes.

*Generation of AAV2 helper capsids encoding mutant capsid proteins*

As the pUCBM21.Cap2 series of plasmids do not contain the AAV2 *rep *gene, in a final cloning step, the wild type and mutant AAV2 *cap* genes were transferred to the AAV vector helper plasmid pAAV2/8(http://www.med.upenn.edu/gtp/vectorcore/Catalogue.shtml#AAVplasmids). For this purpose, pAAV2/8 DNA was cut SmiI and Eco32I and the 4.9-kb digestion product containing the plasmid backbone was purified from agarose gel. Next, the purified plasmid backbone was dephosphorylated using shrimp alkaline phosphatase and the enzyme was inactivated by incubation for 15 min at 65°C. pUCBM21.Cap2, as well as the pUCBM21.Cap2 derivatives containing one, two or three mutations in the *cap *gene was cut with SmiI and the 2.3kb fragments containing the wild type or mutant AAV2 sequences mwere purified from agarose gel. The pAAV2/8 backbone was then combined with each one of the AAV2 *cap* gene-containing fragments to generate pAAV2/2(+/-), pAAV2/2Y444F(+/-), pAAV2/2Y500F(+/-),pAAV2/2Y730F(+/-),pAAV2/ 2Y444500F(+/-),pAAV2/2Y444730F(+/-),pAAV2/ 2Y500730F(+/-) and pAAV2/ 2Y444500730F(+/-). Finally, restriction enzyme digestions were used to select, from these constructs, clones which had the AAV2 *cap* gene inserted in the proper orientation.

## Results

**Figure 2:**
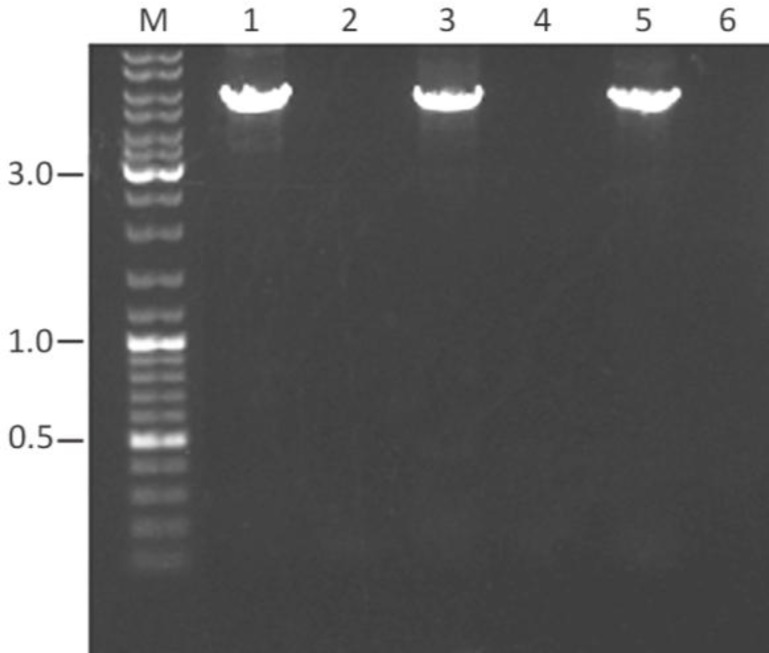
Agarose gel electrophoresis of the pUCBM21.Cap2 amplification products obtained after PCR with primers A001/A002 (lanes 1 and 2), A003/A004 (lanes 3 and 4) or A005/A006 (lanes 5 and 6) in the presence (lanes 1, 3 and 5) or absence (negative controls; lanes 2, 4 and 6) of VELOCITY DNA polymerase. One-tenth of each PCR sample was applied to a 0.8% agarose gel (Tris-acetate-EDTA buffer system) following the removal of proteins, primers and dNTPs by SureClean treatment. Figure shows the PCR products before DpnI treatment. Numbers at the left and right of the gel picture represent sizes in kb. Lane M, GeneRuler DNA Ladder Mix (Fermentas) molecular weight marker

To replace the surface-exposed tyrosine residues at positions 444, 500 and 730 of AAV2 VP1 by phenylalanines, which are chemically similar but non-phosphorylatable, primer pairs A001/A002, A003/A004 and A005/A006 were designed (Table 1). In these primers the tyrosine-encoding TAC codons are replaced by phenylalanine-specifying TTC codons. PCR amplification of pUCBM21.Cap2 with these primer pairs yielded prominent reaction products of 5 to 6 kb (Figure 2, lanes 1, 3 and 5), implying that the whole template DNA had been amplified. 

As expected, PCR amplifications performed in the absence of DNA polymerase did not yield any detectable DNA species following agarose gel electrophoresis (Figure 2, lanes 2, 4 and 6).

**Figure 3:**
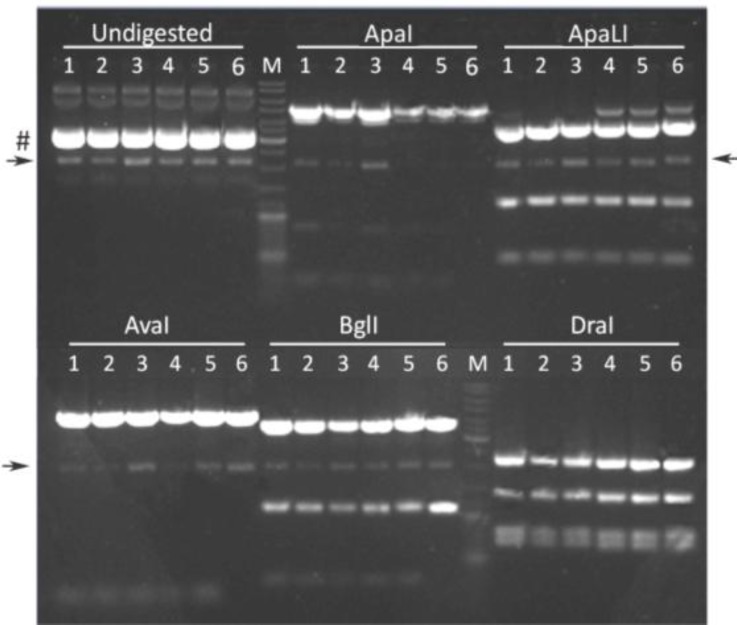
Agarose gel electrophoresis of pUCBM21.Cap2 DNA derivatives after digestion with ApaI, ApaLI, AvaI, BglI or DraI. For comparison, also undigested plasmid DNA was loaded on gel. DNA species indicated by a hashtag and an arrowhead represent supercoiled and denatured plasmid DNA, respectively. Lanes 1 and 2, plasmid pUCBM21.Cap2Y444F; lanes 3 and 4, plasmid pUCBM21.Cap2Y500F; lanes 5 and 6, plasmid pUCBM21.Cap2Y730F; lanes M, GeneRuler DNA Ladder Mix (Fermentas) molecular weight marker

After DpnI treatment to remove the DNA template, 4 μl of each purified PCR sample was introduced into chemocompetent cells of *E. coli *strain GeneHogs, which were subsequently spread on ampicillin-containing LB agar plates and incubated overnight at 37°C. The next day, colonies were visible only on the agar plates corresponding to PCR tubes 1, 3 and 5. Two colonies from each of these plates were used for initiating 200 ml bacterial cultures. The plasmid DNA extracted from the bacteria in the six cultures was analysed by restriction enzyme digestions with ApaI, ApaLI, AvaI, BglI and DraI followed by agarose gel electrophoresis. The predicted sizes of the resulting DNA fragments are indicated in Table 3. As shown in Figure 3, each of the restriction enzymes yielded fragments consistent with the recovery of pUCBM21.Cap2 DNA.

**Table 2:** Nucleotide sequences of primers used for nucleotide sequence analysis. The AAV2 genome sequence was retrieved from GenBank (accession number: AF043303)

Primer	Sequence (5'→3')	AAV2 Genome Position
M13-R	CAGGAAACAGCTATGACCATGA (22 bp)	N/A
A007-F	GCATTGCGATTCCACATGGA (20 bp)	2886-2905
A010-R	GTGCTGTAGCCAAAGTAGTGA (21 bp)	3032-3012
A008-F	GAGGACGTTCCTTTCCACA (19 bp)	3448-3466
A011-F	GCAACAGACAAGCAGCTAC (19 bp)	3959-3977
M13-F	GACGTTGTAAAACGACGGCCAGT (23 bp)	N/A

**Table 3:** Predicted DNA fragment lengths after digestion of plasmid pUCBM21.Cap2 DNA with ApaI, ApaLI, AvaI, BglI or DraI

Restriction enzyme	pUCBM21.Cap2 DNA fragments (bp)
ApaI	4179, 817, 286
ApaLI	3539, 1246, 497
AvaI	4641, 251, 204, 186
BglI	3862, 1118, 302
DraI	2323, 1425, 815, 692, 19, 8

**Figure 4:**
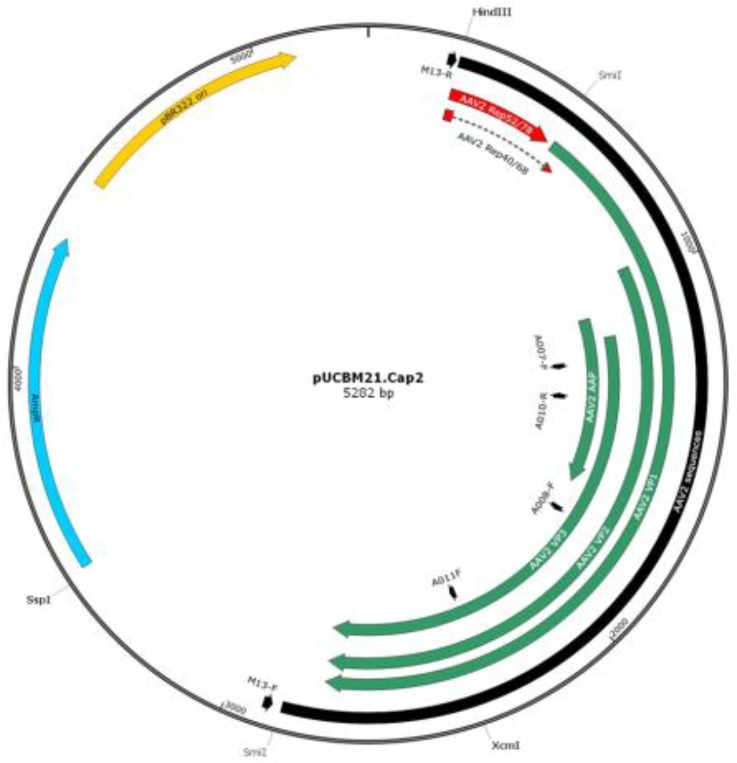
Map of plasmid pUCBM21.Cap2 showing the location of the binding sites for primers M13-R, A007-F, A010-R, A008-F, A011-F and M13-F. Also indicated is the AAV2 sequences (black), relevant restriction enzyme recognition sites, the coding sequences of the structural AAV2 proteins (VP1, VP2 and VP3; green) and of the carboxy-terminal ends of the non-structural AAV2 proteins (Rep78, Rep68, Rep 50 and Rep 40; red), the *Escherichia coli bla *gene conferring resistance to ampicillin (AmpR; blue) and the pBR322 origin of replication (pBR322 ori; yellow)

As none of the restriction endonucleases used to investigate the global structure of the pUCBM21.Cap2 derivatives generated by PCR could make the distinction between wild type and mutant AAV *cap* genes, nucleotide sequence analysis was performed on all six plasmids. Six different primers were used for DNA sequencing (Table 2, Figure 4) to not only check the presence of the desired mutation but also investigate whether the PCR procedure had not introduced any additional unintended mutation(s) in the AAV 2 *cap* gene.

**Figure 5:**
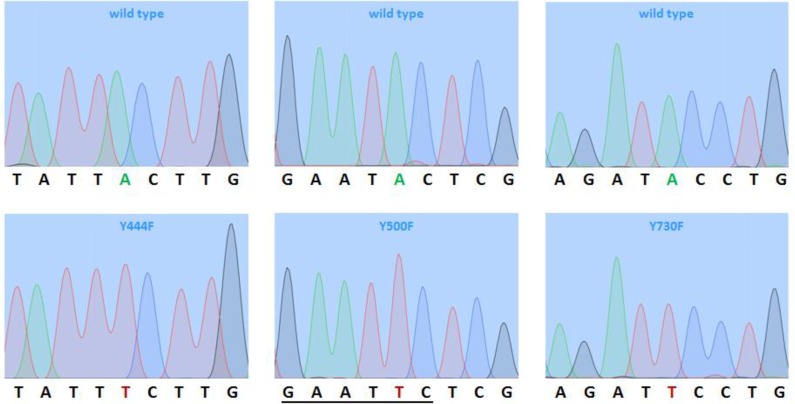
Chromatograms of the nucleotide sequences of wild type pUCBM21.Cap2 and of the pUCBM21. Cap2 mutants pUCBM21.Cap2Y444F, pUCBM21.Cap2Y500F and pUCBM21.Cap2Y730F at the sites of mutagenesis. The chromatograms on the left and in the middle panels were obtained using primer A008-F while those on the right panel were acquired with primer A011-F. Green, blue, black and red peaks correspond to A, C, G and T residues, respectively. The underlined nucleotide sequence represents the EcoRI recognition sequence resulting from the mutagenesis of codon 500 in the AAV2 *cap *gene

The nucleotide sequence analysis confirmed that all six plasmids contained the intended A-T transversions (Figure 5) resulting in the change of tyrosine-encoding TAC codons into phenyalanine-specifying TTC codons. Beside these transversions, no other mutations were detected in the AAV2 *cap *gene of any of the six constructs. Accordingly, the high-fidelity PCR cloning procedure applied in this study allowed us to produce the mutant pUCBM21.Cap2 plasmids pUCBM21.Cap2Y444730F, pUCBM21.Cap2Y500730F and pUCBM21.Cap2 Y444500730F in a single step.

**Figure 6:**
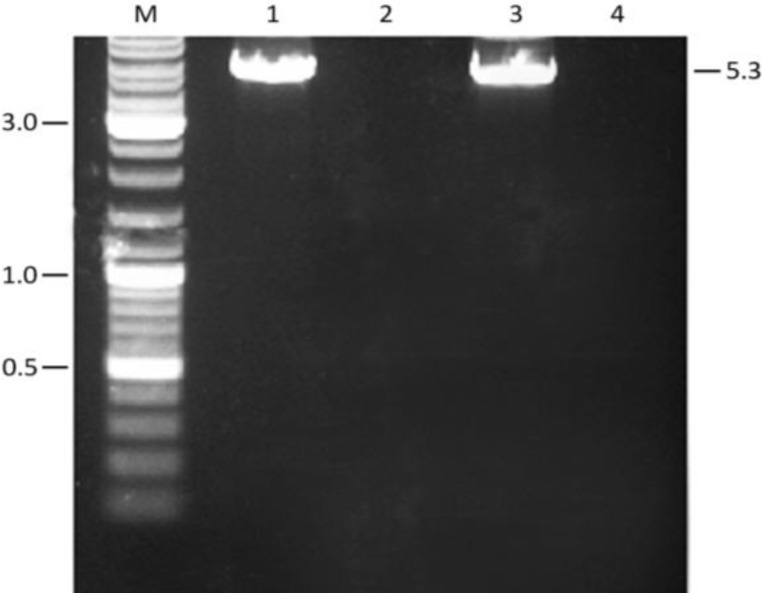
Agarose gel electrophoresis of the PCR products obtained after amplification of pUCBM21.Cap2Y500F with primers A001/A002 (lanes 1 and 2) and pUCBM21.Cap2Y444F with primers A003/A004 (lanes 3 and 4) in the presence (lanes 1 and 3) or absence (negative controls; lanes 2 and 4) of VELOCITY DNA polymerase. One-tenth of each PCR sample was applied to a 0.8% agarose gel (Tris-acetate-EDTA buffer system) following the removal of proteins, primers and dNTPs by SureClean treatment and the degradation of the template DNA by DpnI treatment. Numbers on the left and on the right of the gel picture represent sizes in kb. Lane M, GeneRuler DNA Ladder Mix (Fermentas) molecular weight marker

Due to the lack of convenient restriction enzyme recognition sites between codon 444 and 500 in the AAV2 *cap* gene, it was not possible to generate the double-mutant plasmid pUCBM21.Cap2Y444500F by conventional cloning. Accordingly, another round of PCR mutagenesis was performed with primers A003 and A004 using pUCBM21.Cap2Y444F as DNA template and also with primers A001 and A002 using pUCBM21.Cap2Y500F as DNA template. As shown in Figure 6, both combinations of template and primers yielded amplification products of the correct size in the presence but not in the absence of DNA polymerase.

Restriction enzyme analysis with ApaLI, AvaII, BstXI, DraI and EcoRI was used to verify the integrity and identity of plasmid pUCBM21.Cap2Y444500F. While pUCBM21.Cap2Y444F has two recognition sites for EcoRI, pUCBM21.Cap2Y500F and pUCBM21.Cap2Y444500F possess three recognition sites for this enzyme (Figure 5, Table 4) due to the change of codon 500 from TAC (Y) into TTC (F).

**Figure 7:**
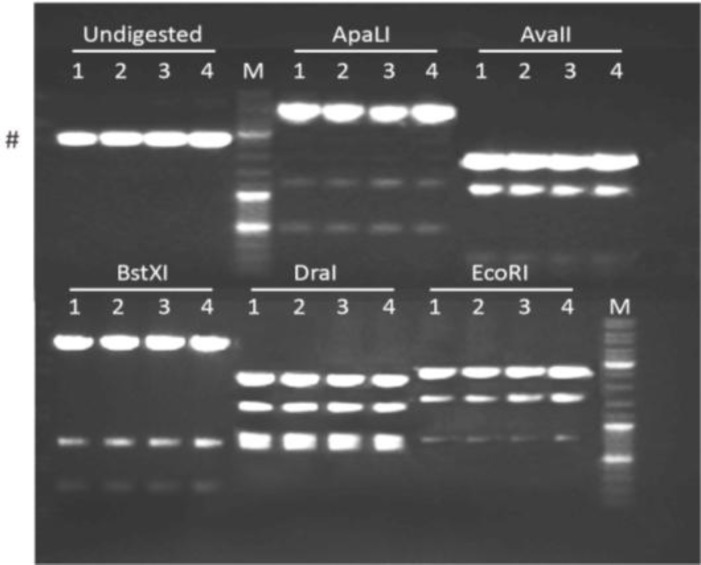
Agarose gel electrophoresis of pUCBM21.Cap2 DNA derivatives after digestion with ApaLI, AvaII, BstXI, DraI or EcoRI. For comparison, also undigested plasmid DNA was loaded on gel. Lanes 1 and 2, plasmid pUCBM21.Cap2Y444500F derived from pUCBM21.Cap2Y444F; lanes 3 and 4, plasmid pUCBM21.Cap2Y444500F derived from pUCBM21.Cap2Y500F; lanes M, GeneRuler DNA Ladder Mix (Fermentas) molecular weight marker. #, supercoiled plasmid DNA

As shown in Figure 7, both cloning strategies yielded plasmids with restriction patterns expected for pUCBM21.Cap2Y444500F. DNA sequence analysis confirmed that AAV *cap* gene codons 444 and 500 had been changed from TAC into TTC for all four plasmid clones and that no inadvertent mutations were introduced during PCR (data not shown).

**Table 4:** Predicted DNA fragment lengths after digestion of plasmid pUCBM21. Cap2Y444F DNA with ApaLI, AvaII, BstXI, DraI or EcoRI. pUCBM21. Cap2Y500F and pUCBM21.Cap2Y444500F yield identical restriction patterns as pUCBM21.Cap2Y444F except for the presence of three instead of two EcoRI cleavage products

Restriction Enzyme	pUCBM21.Cap2Y444F DNA fragments (bp)
ApaLI	3539, 1246, 497
AvaII	1913, 1869, 1130, 222, 123, 25
BstXI	4274, 647, 182, 179
DraI	2323, 1425, 815, 692, 19, 8
EcoRI	2738, 2544 (1712 + 832 in Y500F mutant plasmids)

**Figure 8:**
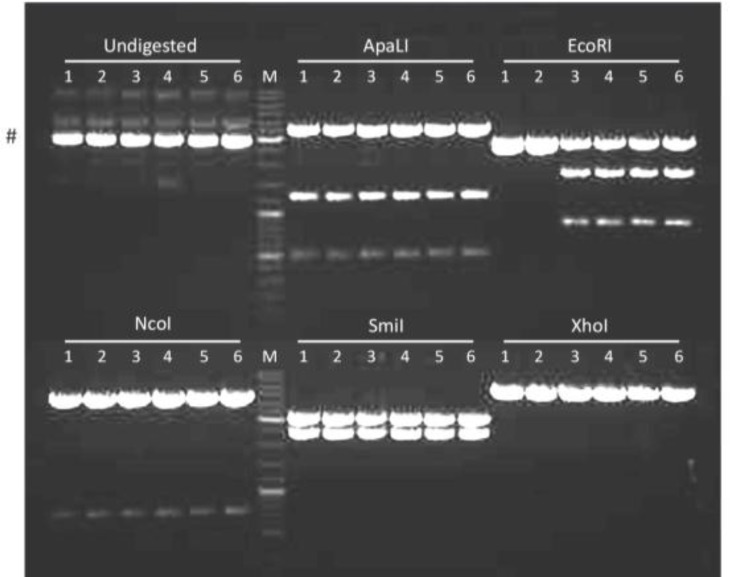
Agarose gel electrophoresis of pUCBM21.Cap2 DNA derivatives after digestion with ApaLI, EcoRI, NcoI, SmiI or XhoI. For comparison, also undigested plasmid DNA was loaded on gel. Lanes 1 and 2, plasmid pUCBM21.Cap2Y444730F; lanes 3 and 4, plasmid pUCBM21.Cap2Y500730F; lanes 5 and 6, plasmid pUCBM21.Cap2Y444500730F; lanes M, GeneRuler DNA Ladder Mix (Fermentas) molecular weight marker. #, supercoiled plasmid DNA

The other double-mutant plasmids (*i.e. *pUCBM21.Cap2Y444730F and pUCBM21.Cap2Y500730F) and the triple-mutant plasmid pUCBM21.Cap2Y444500730F were generated from other pUCBM21.Cap2 derivatives by subcloning using the restriction enzymes XcmI and SspI, which both cut only once in the pUCBM21.Cap2 series of plasmids (Figure 4). Restriction enzyme analysis of the cloning products with ApaLI, EcoRI, NcoI, SmiI and XhoI demonstrated that the proper constructs were obtained (Figure 8).

Finally, the wild type and the mutant AAV2 *cap* genes were inserted into the AAV packaging plasmid pAAV2/8 in place of the AAV serotype 8 *cap *gene as detailed in the Methods section.

## Discussion

There are different PCR-based methods for mutagenesis of double-stranded DNA. Overlap extension PCR, megaprimer PCR, inverted PCR and recombination PCR are some examples of PCR protocols that can be used to introduce mutations in DNA constructs (17-20).

**Table 5:** Predicted DNA fragment lengths after digestion of plasmid pUCBM21. Cap2Y444730F DNA with ApaLI, EcoRI, NcoI, SmiI or XhoI. pUCBM21. Cap2Y500730F and pUCBM21.Cap2Y444500730F yield identical restriction pattern as pUCBM21.Cap2Y44730F except for the presence of three instead of two EcoRI cleavage products

Restriction enzyme	pUCBM21.Cap2Y444730F DNA fragments (bp)
ApaLI	3539, 1246, 497
EcoRI	2738, 2544 (1712 + 832 in Y500F mutant plasmids)
NcoI	4561, 721
SmiI	2959, 2323
XhoI	5096, 186

In this study, a modification of the overlap extension PCR procedure described by Ho and colleagues was used to introduce point mutations in the AAV2 *cap* gene (17). The major differences with the method of Ho *et al *are: (i) the use of a high-fidelity thermostable polymerase instead of the error-prone Taq polymerase to minimize the risk of introducing unintended mutations in the PCR products, (ii) the amplification of an entire plasmid as opposed to only a part of a plasmid to enable the recovery of mutant DNA directly from bacteria and (iii) the use of partially instead of completely overlapping primers (Figure 1) allowing the newly synthesized DNA to serve as template in subsequent PCR cycles (16). Due to the altered primer design, less DNA template is required to obtain sufficient amounts of PCR product making it easier to eliminate the input DNA by DpnI digestion prior to bacterial transformation (16, 21, 22). As a consequence, virtually all bacterial clones obtained after introduction of the DpnI-treated PCR samples in *E. coli *will contain the intended mutation(s). This was confirmed by our results as all ten plasmid clones that were directly derived from PCR products, contained the desired mutations in the AAV2 *cap* gene.

In a preliminary experiment, we compared the ability of AAV2 vectors encoding wild type Cap proteins with AAV2 vectors carrying the three Y→F mutations in their capsids to transduce human myocardial scar cells, neonatal rat cardiac fibroblast and cardiomyocytes. This experiment revealed that both vector types were equally efficient in transducing human myocardial scar cells but that the mutant AAV2 vectors transduced neonatal rat cardiac fibroblast and cardiomyocytes, better (data not shown).

Since the discovery of the knowledge that replacement of certain surface-exposed tyrosine residues in their capsids by phenylalanines renders AAV2 vector less susceptible to degradation (11), researchers have also started to study the effects of mutating surface-exposed lysine, serine and threonine residues on the transduction efficiency of AAV2 vectors. Lysines are the natural targets of ubiquitination and serines and threonines are the only amino acids beside tyrosines with free hydroxyl groups in their side chains that can become phosphorylated. These very recent studies revealed that the performance of AAV2 vectors can also be improved by changing certain surface-exposed serine and threonine residues in non-phosphorylatable amino acids and by replacing specific lysines in the capsid proteins by arginines (23-25). This might provide a rationale for the introduction of additional mutations in the AAV2 *cap* gene using the simple, fast and reliable PCR-based mutagenesis procedure described above. 

## Conclusion

The aim of this paper was to generate AAV2 helper plasmids encoding Cap proteins in which specific surface-exposed tyrosine residues had been changes into phenylalanines to render the AAV2 capsid less susceptible to proteolytic degradation. For this purpose, we used a streamlined PCR-based mutagenesis procedure that is efficient and reliable, takes relatively little time when compared to other mutagenesis protocols and is characterized by low reagent costs.
